# The future of public health: a vision grounded in data and values

**DOI:** 10.1093/eurpub/ckae177

**Published:** 2025-03-25

**Authors:** Sandro Galea

**Affiliations:** Office of the Dean, Washington University School of Public Health, St. Louis, MO, United States

The COVID-19 pandemic galvanized global attention on public health in an unprecedented fashion. More than 7 million people died worldwide as a result of the pandemic, the worst such pandemic in more than 100 years [1]. The pandemic was lived worldwide against a backdrop of issues—climate change, racism, war, authoritarian political movements, and resurgent great power conflict—that all challenged the health of populations. With these challenges in mind, any effort to suggest a future of public health must do so cautiously.

With all due caution then, I offer a vision for the future of public health grounded in a balance of empiric and value-based concerns. Data should help focus where we dedicate our attention and resources. Values keep our efforts rooted in our core mission. Together, data and values should be the bricks and mortar of public health, helping us build toward the future of the field.

I start the data. There are five key forces that will shape health, and should be at the heart of the work of public health, in the coming decades: infectious disease, urbanization, climate change, our engagement with mental and behavioral health, and health inequities.

First, the COVID-19 pandemic was a dramatic example of how a virus can take hold among the global population. As devastating as COVID-19 was, however, it could have been far worse. COVID-19 was highly transmissible but, compared with past pandemics, not very lethal. There is no reason to think that the next pandemic will not combine the transmissibility of COVID with the lethality of a far deadlier pathogen. Yet the specter of such a pandemic is not the only reason we should concern ourselves with the threat of infectious disease. There were 1307 epidemic events between 2011 and 2017 [2]. These include outbreaks of cholera, Zika, meningitis, and influenza. In the age of climate change, urbanization, and global travel, such threats have new avenues to spread. We need to address this challenge not just by working to develop new and better therapeutics—as important as these are. We must also engage with the forces that drive epidemics by dealing with the socioeconomic inequities that create pockets of poor health in society. When forces like poverty, racism, inequality, and stigma keep health out of reach for certain groups, we are all vulnerable. We cannot be healthy until we are all healthy; this is particularly true in the context of infectious disease.

Second, in 2007, the UN estimated that, for the first time, more people lived in cities than in rural areas [3]. Today, over half of the global population lives in urban areas [3]. By 2050, that figure is projected to jump to over two-thirds of the global population. This trend has profound implications for health. On one hand, there is much about urbanization that is good for health. People tend to move to cities when they become better-off and living standards in urban areas are generally better than in rural ones. On the other hand, cities present unique challenges for health. These include exposure to pollution, lack of access to parks and green spaces, the concentration of poverty and crime in parts of cities, and challenges to mental health that can come with noise and a sense of urban isolation. These influences mean that how we design cities will have a significant, even decisive effect on the future of health. Will we shape cities in ways that amplify their worst features? Or will we design them with an eye toward health? The task of public health will be to help ensure urban populations have access to the full range of resources that shape health. None of this aims to negate the very real challenges of living in rural areas, and indeed there should be space for the future of public health to encompass the concerns of rural living. It does, however, recognize trends toward urbanization and toward population density in urban environments that should merit the attention of public health going forward.

Third, the health of our planet is central to the health of the public. That seems self-evident, yet it has been a long road to creating an awareness of climate change as a public health issue. Global temperatures have risen significantly over the last 150 years [4]. We have started seeing the consequences of this change in extreme weather events, wildfires, and changes in animal migration patterns. The distribution of these effects has so far been deeply unequal, with wealthy, industrialized countries generating most of the planet’s carbon and poorer countries with small carbon footprints facing the brunt of climate change. But these consequences will not stay compartmentalized forever. It is just a matter of time before the rest of the world feels the full effects of climate change, in climate-driven mass migration and in the direct effects of extreme weather and rising sea levels. Addressing climate change means a data-informed combination of adaptation and mitigation. We need a plan to cut carbon emissions while engaging with the effects of climate change that are already here. It is on us to create communities that can withstand the effects of rising sea levels and extreme weather. We also need to make sure that wealthy countries do their fair share to address the consequences of their longstanding neglect of climate change by using their resources to support the populations that currently face the worst of this crisis. We should also note that some of the core needs of public health, including, e.g. data-intensive work, push on energy needs globally. That does not need to minimize our commitment to planetary health and mitigation of the consequences of high-energy demands, but rather should encourage us to be part of the solution, entwined, as public health should be, with the real world.

Fourth, one of the central challenges of the COVID moment was its effect on our collective mental and behavioral health. Mood–anxiety disorders increased globally. Depression in the USA tripled during the pandemic [5]. The risk of depression during the pandemic was greater for those who lacked social and economic assets. This speaks to the importance of engaging with the socioeconomic drivers of mental health, just as we do with physical health. Mental health is public health. Yet we have not always given mental health the attention it deserves. This is in part due to stigma about discussing mental health. It is also seen in the lack of resources we devote to supporting mental health. Just using the USA as an example, the fruits of this disinvestment are in the over 40 000 Americans who died by suicide in 2020 [6], increased substance use [7], and an annual cost for untreated mental illness that runs into the billions [8]. Creating a healthier future means supporting mental health by both investing in mental health care and addressing the structural drivers of mental illness.

Fifth, if there is a through-line to these foundational forces, it is the challenge of inequality and inequity. We live in a world of health haves and have-nots. In this world, a fortunate few have the resources to live healthy lives just about no matter what. Even during a crisis like a pandemic, their socioeconomic resources let them maintain a level of comfort and health. They can avoid the consequences of climate change by simply moving away from its worst effects. They can get the best out of urbanization by affording to live in the choicest parts of cities. They never lack the resources that support physical and mental health. Meanwhile, those with less privilege and fewer resources live shorter, sicker lives. This is perhaps clearest when it comes to global life expectancy, where there is a vast gulf between countries with the most resources and countries with the least. We also see it in the inequality of climate change—where 20 of the richest billionaires are estimated to emit up to 8000 times more carbon than the billion poorest people [9]. In the USA, we see inequality in everything from racial health inequities to the 10-year gap between the life expectancy of the richest and poorest Americans [10]. As living standards improve and as technology becomes ever more advanced, there will be a temptation to feel like our work is done, like utopian progress has been made. It will be easy to miss the many for whom this is not the reality. We must make sure this does not happen. That will require a clear-eyed focus on health inequity in everything we do, contributing to the design of efforts and programs that aspire not only to advance health but also to ensure that there is no one left behind on health through active and intentional design.

I identify these challenges as fundamental, seeing them as five dominant concerns that should occupy the focus of public health in the coming decades. I note that this does not include other forces—i.e. population aging, migration, and the rapid advance of new technologies like artificial intelligence—that are also gathering steam, that will undoubtedly be important forces shaping the health of populations throughout the twenty-first century, and should as such also be in our remit. But a consideration of the data that inform the role of these forces will not, by itself, be sufficient for public health to act on these issues. For that, we need values. Values can guide our focus and help ensure the integrity of our efforts. They keep us grounded in the best traditions of public health. With this in mind, I suggest the following value-based affirmations to advance a vision of a healthier world in the years to come.

First, we are committed to a diverse and inclusive public health community. In public health, we serve a diverse range of populations. The ranks of public health should reflect these populations. For this reason, we should prioritize, always, ensuring that all have a seat at the table and that all are included in the public health conversation.

Second, we are committed to equity. Sometimes what we think of as equality can mask deep, persistent unfairness. Equity reflects a leveling of the playing field, so all can access the resources necessary for health.

Third, we value a plurality of ideas and perspectives grounded in truth. Diversity does not just mean diversity of identity. It also means diversity of thought and opinion. We should welcome engagement with ideas that challenge us to sharpen our thinking in pursuit of our goals. Public health is deeply informed by the legacy of the scientific revolution, the enlightenment, and comparable forces across the world. At the heart of this legacy is the reasoned pursuit of truth. Truth, more than anything—more than narrative or political ideology—should be the lodestar of our efforts. This recognizes that public health often involves tradeoffs. During COVID-19, we had to navigate a complicated balance between the virus on one hand and the socioeconomic costs of lockdowns on the other. We did not always strike this balance well, struggling to balance the needs of societies and economies with the imperatives of infectious disease control. Going forward, we must be more willing to engage with the tradeoffs inherent in choices about health.

Fourth, we are committed to human rights, emphasizing freedom and autonomy. Health is inseparable from freedom and human dignity. Public health must be committed to a vision of human rights that supports these conditions. Whenever we see human rights violated, even—especially—when it is being done in the name of health, we have a responsibility to speak out, to bear witness.

Fifth, we celebrate what unites us rather than what divides us. Health is perhaps the ultimate unifying principle. All of us, no matter who we are, want to be healthy. Public health is uniquely positioned to keep focus on this most uniting of ideals. In an age of division, this is among the most important contributions we as a field can make.

I suggest that these values can help us balance an empiric focus on data with a humane, moral vision for a healthier world. These values also, combined with data, point to a picture of what public health can face in the years ahead and how we can act to better live up to our mission to create a healthier world.
